# The Brainstem-Vermis and Brainstem-Tentorium Angles in the Fetus: A Study of Their Reproducibility by Fetal Magnetic Resonance Imaging and Their Evolution Along the Gestation

**DOI:** 10.3389/fmed.2022.878906

**Published:** 2022-05-26

**Authors:** Laura Joigneau Prieto, Yolanda Ruiz, Laura Pérez, Coral Bravo, Alejandra Aguado, Melchor Alvarez-Mon, Miguel A. Ortega, Carlos Marín, Juan De León-Luis

**Affiliations:** ^1^Department of Radiology, University Hospital Gregorio Marañón, Madrid, Spain; ^2^Department of Public and Maternal and Child Health, School of Medicine, Complutense University of Madrid, Madrid, Spain; ^3^Health Research Institute Gregorio Marañón, Madrid, Spain; ^4^Department of Obstetrics and Gynecology, University Hospital Gregorio Marañón, Madrid, Spain; ^5^Ramón y Cajal Institute of Healthcare Research (IRYCIS), Madrid, Spain; ^6^Department of Medicine and Medical Specialties, Faculty of Medicine and Health Sciences, University of Alcalá, Alcalá de Henares, Madrid, Spain; ^7^Immune System Diseases-Rheumatology, Oncology Service an Internal Medicine (CIBEREHD), University Hospital Príncipe de Asturias, Alcala de Henares, Spain; ^8^Department of Cancer Registry and Pathology, Hospital Universitario Principe de Asturias, Alcala de Henares, Spain

**Keywords:** posterior fossa fluid collections, BV angle, BT angle, prenatal ultrasound, fetal MRI

## Abstract

**Aim:**

To assess the reproducibility of brainstem-vermis (BV) and brainstem-tentorium (BT) angles measured by fetal Magnetic Resonance Imaging (MRI) during second half of pregnancy in normal and abnormal fetuses. Secondly, to assess reproducibility of two alternative methodologies to measure the brainstem-tentorium angle (BT1 and BT2) proposed by our group that could be more reliable in fetuses with posterior fossa fluid collection (PFFC) anomalies. Finally, to describe the evolution of BV and BT angles along gestation in normal fetuses.

**Methods:**

We conducted a cross-sectional study of BV and BT angles obtained by MRI performed at our center, in 22 fetuses with PFFC and 8 fetuses without PFFC to calculate both angles’ reproducibility and the correlation between them and the gestational age.

**Results:**

We found good interobserver reproducibility for the BV, BT1 and BT2 angles (Intraclass correlation coefficient: 0.98; 0.89 and 0.88 for each of these angles, with *p* < 0.001). In patients with PFFC the BT angle could not always be measured. BT angle presented a positive relationship with gestational age (*p* = 0.002) but BV angle stayed stable. The measurements of BV, BT1, and BT2 angles can be reliably performed by MRI with good interobserver reproducibility.

**Conclusion:**

BV angle stays stable during pregnancy, whereas BT angle tends to augment with gestational age.

## Introduction

Prenatal ultrasound is currently the main diagnostic tool to detect fetal malformations. However, fetal magnetic resonance imaging (MRI) is increasingly being used for the evaluation of the central nervous system (CNS) (1–3), as it can provide detailed information about normal and abnormal neuroanatomy (4) and therefore identify other CNS malformations that are typically not detected by ultrasound. Among the CNS malformations, fetal posterior fossa fluid collections (PFFC) associated with upward displacement of the tentorium and rotation of the cerebellar vermis are not very frequently found but have a highly variable neurological prognosis. They can range from normal variants to severe anomalies (5–7): Blake’s pouch cyst, megacisterna magna, arachnoid cyst, vermian hypoplasia and Dandy-Walker malformation.

The differential diagnosis of these entities is mainly based upon the rotation of the cerebellar vermis relative to the brainstem [brainstem-vermis (BV) angle] and the level of the tentorium insertion relative to the brainstem [brainstem-tentorium (BT) angle]. Both angles were proposed by Ghi et al. (8). They reported that these two angles could be reproduced by ultrasound at midtrimester scan in normal fetuses, however, BV angle seems to be more accurate than the BT angle in the evaluation of the PFFC (9, 10). We previously reported that the measurement of the BT angle could be challenging in some cases of PFFC (11). This could be one of the reasons why BT seems less accurate in the evaluation of the PFFC.

The first purpose of this study is to assess the reproducibility of the BV and BT angles measured by fetal MRI during the second and third trimester of pregnancy in normal and abnormal fetuses. The second aim of this study is to assess reproducibility of two alternative methodologies to measure the BT angle by fetal MRI -BT1 and BT2 angles- proposed by our group, that could be more reliable in fetuses with PFFC anomalies. Finally, we will describe the evolution of the BV and BT angles along the gestation in normal fetuses.

## Materials and Methods

This was a cross-sectional observational study carried out in our Fetal Medicine Unit reviewing the fetal MRI performed at our center. Patients were recruited consecutively from a cohort of pregnant women referred for fetal MRI evaluation due to the suspicion of fetal or placental anomalies visualized by ultrasound. All pregnancies were dated during a first-trimester ultrasound.

In order to assess the reproducibility of the BV and BT angles measured by fetal MRI, we included all the cases of PFFC (*n* = 22) as well as a sample of fetuses with normal posterior fossa imaging study (*n* = 8). We assessed the reproducibility of the BT angle measured with three different methodologies: the one described by Ghi et al. (8) (that we will call from now BT angle), the BT1 angle and BT2 angle, both previously described by us (11). The measurements were performed by two observers.

Finally, in order to assess the relationship between the BV and BT angles and the gestational age during the second and third trimester, we selected a group of 28 fetuses with normal posterior fossa.

Written informed consent was obtained from all the patients before the MRI. The study was approved (March, 2017) by the Clinical Research Ethics Committee of the Gómez-Ulla-UAH Hospital (37/17).

### Fetal Magnetic Resonance Imaging

The exams were performed in the Department of Pediatric Radiology of our hospital on a 1.5 Tesla (T) Philips Intera system (Best, The Netherlands). The patients were placed in either supine or partial left decubitus position. A small flex four-channel phased-array coil was placed around the maternal abdomen. A three-plane rapid localizer acquisition was performed to ensure the correct positioning of the head of the fetus in the center of the coil and to allow selection of the most amenable section for the first sequence. Axial, coronal and sagittal Balanced Turbo Field Echo (bTFE) sequences, serving each one as a scout for the next one, were obtained of the whole fetal brain, with sections of 4 mm without gap and a field of view (FOV) as small as possible in each case. MRI parameters were: repetition time = 4.2 ms; effective echo time = 2.1 ms; echo train length = 256; number of excitation = 1; matrix = 192 × 256; acquisition time = 18–25 s.

The fetal MRI were read by a 15-year experience radiologist (YR), who selected a cerebral midsagittal plane where the pons, the vermis and the tentorium could be clearly visualized to perform the measurements of the angles.

### Measurement of the Brainstem-Vermis and Brainstem-Tentorium Angles

**FIGURE 1:**
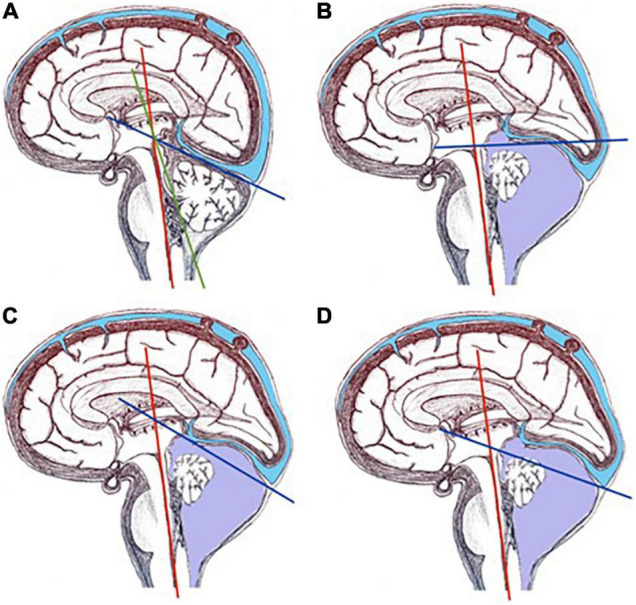
Representation of BV and BT angles in a normal fetus and in a fetus with PFFC. **(A)** Represents BV (red and green lines) and BT (red and blue lines) angles in a normal fetus. **(B–D)** Represent BT angle in a fetus with PFFC: **(B)** is the angle as described by Ghi et al. (8), from the quadrigeminal plate following the tentorium surface; **(C)** represents the BT1 angle, defined with the line that goes from the upper insertion of the tentorium down to its insertion in the occipital bone; **(D)** depicts the BT2 angle, defined with the line that goes from the quadrigeminal plate to the insertion of the tentorium in the occipital bone.

Measurements were performed by YR and LJ, and were blinded to each other. The BV and BT angles were both measured in the midsagittal plane previously selected (Figure 1), and were defined, according to Ghi et al. (8) previous description, as follows. The BV angle is the angle formed by a line tangential to the dorsal part of the brainstem and a line starting from the tip of the pons and crossing the lower edge of the cerebellar vermis. The BT angle is the angle formed by a line tangential to the dorsal part of the brainstem and a line starting from the upper limit of the quadrigeminal plate and following the tentorial surface down to the occipital bone. As pictured in Figure 1, in patients with highly distorted posterior fossa anatomy, the tentorial surface can be difficult to follow with a single line. For this reason, we have proposed two other different ways of measuring the BT angle (11). The BT1 angle is the angle formed by a line tangential to the dorsal part of the brainstem and a line starting from the posterior insertion of the tentorium at the occipital bone up to the anterior insertion of the tentorium. In the BT2 angle, the line that defines the tentorium starts at the posterior insertion of the tentorium at the occipital bone and goes up to the quadrigeminal plate.

### Statistical Analysis

Interobserver agreements for the BV, BT, BT1, and BT2 angles measurements were calculated by the intraclass correlation coefficients (ICCs) and 95% confidence intervals. The agreement was interpreted according to Landis and Koch’s criteria as poor (ICC < 0.21), discrete (ICC: 0.21–0.40), moderate (ICC: 0.41–0.60), good (ICC: 0.61–0.80), and excellent (ICC > 0.80). We illustrated interobserver agreement using Bland-Altman analysis (12).

The correlation between the BV and BT angles and the gestational age was assessed with linear regression analysis. A *p*-value of less than 0.05 was considered to be significant.

We used the SPSS software package (Statistical Product and Services Solutions, version 21.0, SPSS Inc., Chicago, IL, United States).

## Results

**FIGURE 2:**
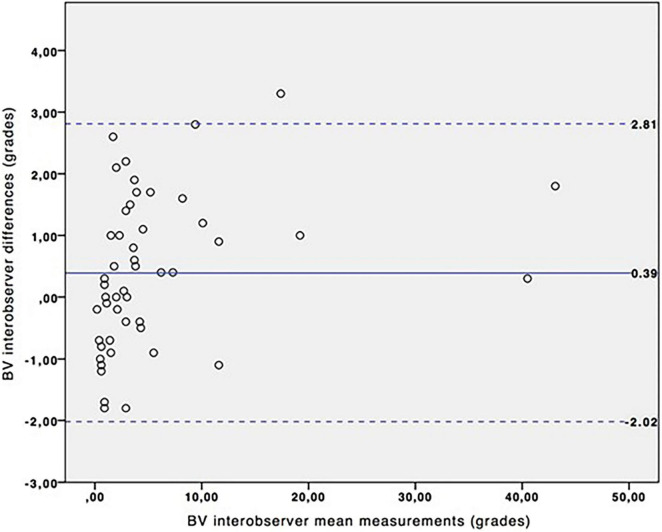
Interobserver variation for BV angle measurements. Bland-Altman plot for BV angle. Continuous blue line represents the mean of the interobserver differences and the dashed lines represent the 95% confidence interval.

**FIGURE 3:**
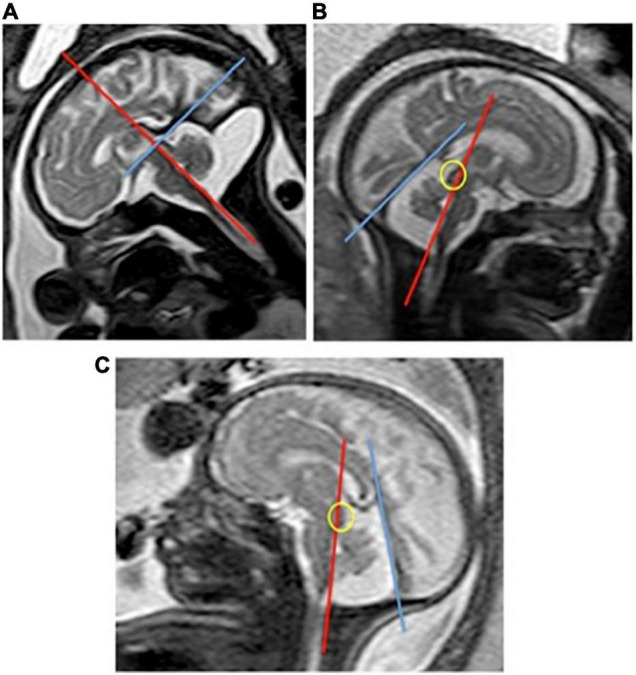
Fetal MRI showing difficulties in the measurement of the BT angle. **(A)** Shows a fetal MRI where the tentorial surface is irregular due to the PFFC. The line that goes from the quadrigeminal plate and follows the tentorium does not represent the tentorial insertion in the occipital bone. **(B,C)** Show two examples of fetuses where it is not possible to draw a line that would go from the quadrigeminal plate (circle) following the tentorial surface.

**FIGURE 4:**
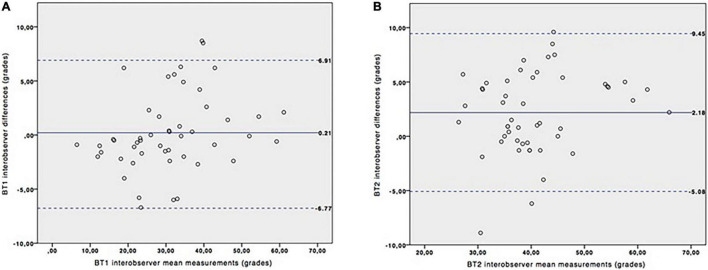
Interobserver variation for BT1 and BT2 angles measurements. Bland-Altman plots for BT1 **(A)** and BT2 **(B)** angles. Continuous blue line represents the mean of the interobserver differences and the dashed lines represent the 95% confidence interval.

Table 1 shows the MRI final diagnosis of the fetuses included in the interobserver reproducibility study as well as the gestational age at which the study was performed (from 21 to 36 weeks of gestation, mean: 31.06). The BV angle was successfully measured in all cases. Table 2 and Figure 2 show good interobserver reproducibility for the BV angle. It was not possible to perform any statistical analysis for the measurement of the BT angle since it could not be measured in almost half of the fetuses (measured in 18 out of 30 patients). This difficulty was related to two facts: first, the quadrigeminal plate was frequently not aligned with the tentorium; and second, the tentorial surface was not always a “*straight*” line (Figure 3) and it was therefore difficult to trace a line following the tentorial surface. Both BT1 and BT2 angles were measured in all the fetuses, and showed good reproducibility (Table 2 and Figure 4), although the BT2 angle was slightly wider than the BT1 angle.

**TABLE 1:** Cases used for the interobserver agreement study.

Case number	GA (weeks)	Magnetic resonance imaging diagnosis
1	31	Megacisterna magna
2	25	Cerebellar hypoplasia and arachnoid cyst
3	36	Arachnoid cyst
4	31	Arachnoid cyst
5	35	Arachnoid cyst
6	30	Arachnoid cyst
7	31	Megacisterna magna
8	30	Megacisterna magna
9	29	Megacisterna magna
10	32	Megacisterna magna
11	36	Cerebellar hypoplasia and arachnoid cyst
12	33	Megacisterna magna
13	35	Megacisterna magna
14	35	Megacisterna magna
15	30	Megacisterna magna
16	34	Megacisterna magna
17	32	Arachnoid cyst
18	34	Arachnoid cyst
19	36	Arachnoid cyst
20	33	Arachnoid cyst
21	21	Cerebellar hypoplasia
22	32	Cerebellar hypoplasia and pons hypoplasia
23	26	No CNS anomalies
24	31	Cerebral morphometry 4 weeks less than expected
25	35	Rabdomyoma of the left ventricle.
26	31	Unilateral ventriculomegaly
27	31	Ventriculomegaly
28	24	Ventriculomegaly
29	22	Mild unilateral ventriculomegaly
30	34	Mild unilateral ventriculomegaly

*Gestational age (GA) at which the fetal MRI was performed. The dashed line represents the limit between cases with PFFC and cases with no posterior fossa anomalies.*

**TABLE 2:** Interobserver concordance of the measurements of BV, BT, BT1, and BT2 angles.

	First observer	Second observer	ICC and 95% CI	*p*
BV angle (mean ± *SD*)	7.62 ± 10.81	6.78 ± 10.74	0.98 (0.97–0.99)	<0.001
BT angle (mean ± *SD*)	-	-	-	-
BT1 angle (mean ± *SD*)	32.39 ± 13.60	32.71 ± 12.87	0.89 (0.78–0.95)	<0.001
BT2 angle (mean ± *SD*)	43.83 ± 10.90	39.12 ± 11.52	0.88 (0.76–0.94)	<0.001

*Means of BV and BT angles measurements performed by YR and LJ, with the interobserver correlation index and its significance with 95% confidence interval.*

*ICC, Intraclass correlation coefficient; CI, confidence interval.*

**FIGURE 5:**
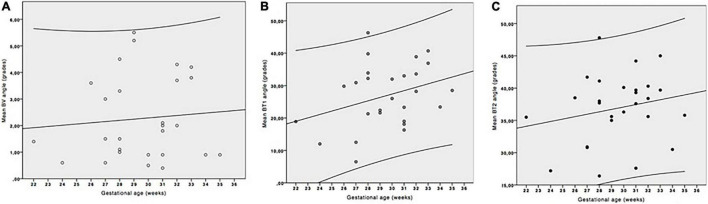
Evolution of the brainstem vermis (BV) **(A)** and brainstem-tentorium (BT) **(B)** angles with gestational age (GA). **(C)** Evolution of the BV (empty dots) and BT (black filled dots) angles with gestational age. Only BT angle shows positive correlation with gestational age and predictive mean of BT angle (grades) = 1.20 × GA (weeks)-2.67; *R*^2^ = 0.30; *p* = 0.002.

The study of the evolution of the BV and BT angles along the gestation was performed from 22 to 36 weeks of gestation (mean: 30). We found that the BV angle did not vary with gestational age (Figure 5A), whereas the BT angle tended to increase with the gestational age (Figures 5B,C) following the equation: Predicted mean of BT (grades) = 1.20 × GA (weeks)-2.67; *R*^2^ = 0.30; *p* = 0.002.

## Discussion

Our results prove that a quantitative assessment of the posterior fossa by measuring angles can be performed by fetal MRI. It has been previously published that the BV and BT angles could be reproducible by ultrasound in normal fetuses from 20 to 21 weeks of gestation (8). We have proven that the BV angle is also highly reproducible in fetal MRI, in fetuses from 21 to 36 weeks of gestation. However, due to the difficulties that we found measuring the BT angle with the methodology described by Ghi et al. (8) in fetuses with PFFC, we could not perform interobserver analysis for this angle. The difficulty to measure the BT angle, both by ultrasound and MRI, has been previously described (11, 13). The two proposed alternatives, BT1 and BT2 angles, have both proven to be more reliable and reproducible, even in fetuses with a highly distorted posterior fossa (Table 2).

The clinical utility of BV and BT angles measured by ultrasound has been previously described by Adamsbaum et al. (5) in fetuses with PFFC between 19 and 28 weeks: the BV angle discriminated accurately between Blake’s pouch cyst, vermian hypoplasia and Dandy-Walker malformation, whereas the BT angle showed overlapping between the groups limiting its diagnostic value. This overlapping could be related to the increase of the BT angle with gestational age. On the other hand, the stability of the BV angle along pregnancy allows its comparison between anomalies without the interference of the gestational age. McKinnon et al. (14) found similarly, that the tegmento-vermian angle measured by MRI remains unchanged with gestational age, whereas measurements of the tentorial angle are correlated with gestational age.

Even though we did not use BV and BT angles measured by MRI to classify our patients’ PFFC, BT1 or BT2 angles could prove to be useful for this objective.

Other measurements have been previously described to objectively assess the PFFC by fetal MRI (15–17). Mckinnon et al. (14) have reviewed all these measurements in a systematic review published in 2020, identifying those that could be useful in the assessment of PFFC. Despite all the previous publications, we propose two easy and reproducible measurements to calculate the brainstem-tentorium angle, (BT1 and BT2 angles), that could be used, not only in MRI, but also in ultrasound. The advantage of these two new angles is that they can be easily traced not only in fetuses with normal posterior fossa, but also in those that have a highly distorted tentorium. We propose these new methodologies to be used in future studies to prove their clinical usefulness, or to use the BV angle alone to categorize PFFC if the BT1 and BT2 angles prove to be less accurate than the BV angle as the BT angle has proven to be.

The small sample used is the main limitation of this study. However, the findings are still interesting since we were able to prove interobserver reproducibility of the measurements in fetal MRI, as well as describe the relationship of the angles with gestational age in normal fetuses. It would be advisable to perform future studies that would include a larger number of patients and include all the possible anomalies causing a PFFC.

## Conclusion

In conclusion, our results suggest that the measurement of BV, BT1 and BT2 angles can be reliably performed by MRI, with good interobserver reproducibility; and even though the BV angle stays stable during the pregnancy, the BT angle tends to increase with gestational age. Future studies are needed in order to assess the utility of BT1 and BT2 angles measured by fetal MRI in the cases of PFFC.

## Data Availability Statement

The raw data supporting the conclusions of this article will be made available by the authors, without undue reservation.

## Ethics Statement

We obtained the approval from the local ethical committee for the study (37/17 March 2017). Written informed consent to participate in this study was provided by the participants’ legal guardian/next of kin.

## Author Contributions

All authors listed have made a substantial, direct, and intellectual contribution to the work, and approved it for publication.

## Conflict of Interest

The authors declare that the research was conducted in the absence of any commercial or financial relationships that could be construed as a potential conflict of interest.

## Publisher’s Note

All claims expressed in this article are solely those of the authors and do not necessarily represent those of their affiliated organizations, or those of the publisher, the editors and the reviewers. Any product that may be evaluated in this article, or claim that may be made by its manufacturer, is not guaranteed or endorsed by the publisher.
